# Evaluation of Intrinsic Charge Carrier Transport at Insulator-Semiconductor Interfaces Probed by a Non-Contact Microwave-Based Technique

**DOI:** 10.1038/srep03182

**Published:** 2013-11-11

**Authors:** Yoshihito Honsho, Tomoyo Miyakai, Tsuneaki Sakurai, Akinori Saeki, Shu Seki

**Affiliations:** 1 Department of Applied Chemistry, Graduate School of Engineering, Osaka University, 2-1 Yamadaoka, Suita, Osaka 565-0871, Japan

## Abstract

We have successfully designed the geometry of the microwave cavity and the thin metal electrode, achieving resonance of the microwave cavity with the metal-insulator-semiconductor (MIS) device structure. This very simple MIS device operates in the cavity, where charge carriers are injected quantitatively by an applied bias at the insulator-semiconductor interface. The local motion of the charge carriers was clearly probed through the applied external microwave field, also giving the quantitative responses to the injected charge carrier density and charge/discharge characteristics. By means of the present measurement system named field-induced time-resolved microwave conductivity (FI-TRMC), the pentacene thin film in the MIS device allowed the evaluation of the hole and electron mobility at the insulator-semiconductor interface of 6.3 and 0.34 cm^2^ V^−1^ s^−1^, respectively. This is the first report on the direct, intrinsic, non-contact measurement of charge carrier mobility at interfaces that has been fully experimentally verified.

Interfaces play an important role in organic electronics from both a structural and functional point of view. For example, material/substrate and material/air interfaces are known to cause molecules to adopt different orientations from those inside the bulk material^1^,^2^,^3^,^4^. Interfaces between *p*- and *n*-type semiconductors have a strong influence on the photovoltaic response^5^,^6^,^7^. In most electronic devices, charge carriers are injected or extracted through metal/semiconductor interfaces during device operation. In particular, organic field-effect transistors (OFETs)^8^,^9^,^10^, one of the most practical device applications of organic semiconductors, rely highly on interfacial phenomena because even charge-carrier transport takes place at insulator/semiconductor interfaces rather than in the bulk. In fact, the hole and electron mobility in OFETs was found to be extremely sensitive to the microscopic morphology of the films at the interfaces^11^,^12^. Although improvement of OFET performance is awaited, tools for analyzing interfacial carrier transport are still undeveloped. Limited examples include the quantification of electronic energy levels of molecules at material surfaces or interfaces by X-ray photoelectron spectroscopy (XPS) or ultraviolet photoelectron spectroscopy (UPS) measurements. Another unique approach was reported by Marumoto and co-workers, who used field-induced electron spin resonance (FI-ESR) to evaluate the spin density distribution of charge carriers at pentacene–SiO_2_ interfaces^13^,^14^. Other studies focused on charge-carrier motion at interfaces, and used organic insulators such as poly(dimethylsiloxane) (PDMS), poly(methylmethacrylate) (PMMA) and self-assembled monolayers (SAM) to control the interfacial morphology^15^,^16^,^17^. Building on this background, we report a novel technique named field-induced time-resolved microwave conductivity (FI-TRMC) for evaluating charge carrier mobility at the interface between insulators and semiconductors without grain boundary effects (Fig. 1). This newly-developed measurement system involves charge carrier generation at insulator-semiconductor interfaces by applying a gate bias and evaluation of carrier mobilities through microwave probing, which potentially clarifies the effect of insulators on the intrinsic mobility of charge carriers in OFETs.

Microwave-based techniques have been recently developed as noncontact evaluation methods to evaluate intrinsic charge carrier mobilities. In a pioneering work, Warman and co-workers reported the pulse radiolysis time-resolved microwave conductivity (PR-TRMC) technique, where radiation-induced charge carriers are made to resonate using a microwave field. Using this method, we evaluated nm-scale motion of charge carriers, obtaining the sum of the local-scale mobility Σ*μ* ( = *μ*_hole_ + *μ*_electron_)^18^,^19^. After that, several researchers including us developed the flash photolysis time-resolved microwave conductivity (FP-TRMC) technique, where charge carriers are generated by photoinduced charge-separation processes^20^,^21^,^22^,^23^,^24^. Use of photoexcitation process allowed us to cover larger variety of materials and to determine their carrier mobility using quite a small amount of samples. However, because of the radiation or photoexcitation induced ionization mechanisms involved, both holes and electrons are generated together, which makes it difficult to differentiate the contributions from the individual hole and electron mobilities. For that reason, the FI-TRMC system is noteworthy, since the system clarifies the contribution of hole or electron conduction at the interfaces by applying a positive or negative bias selectively.

## Results

### Experimental setup

**Figure 2:**
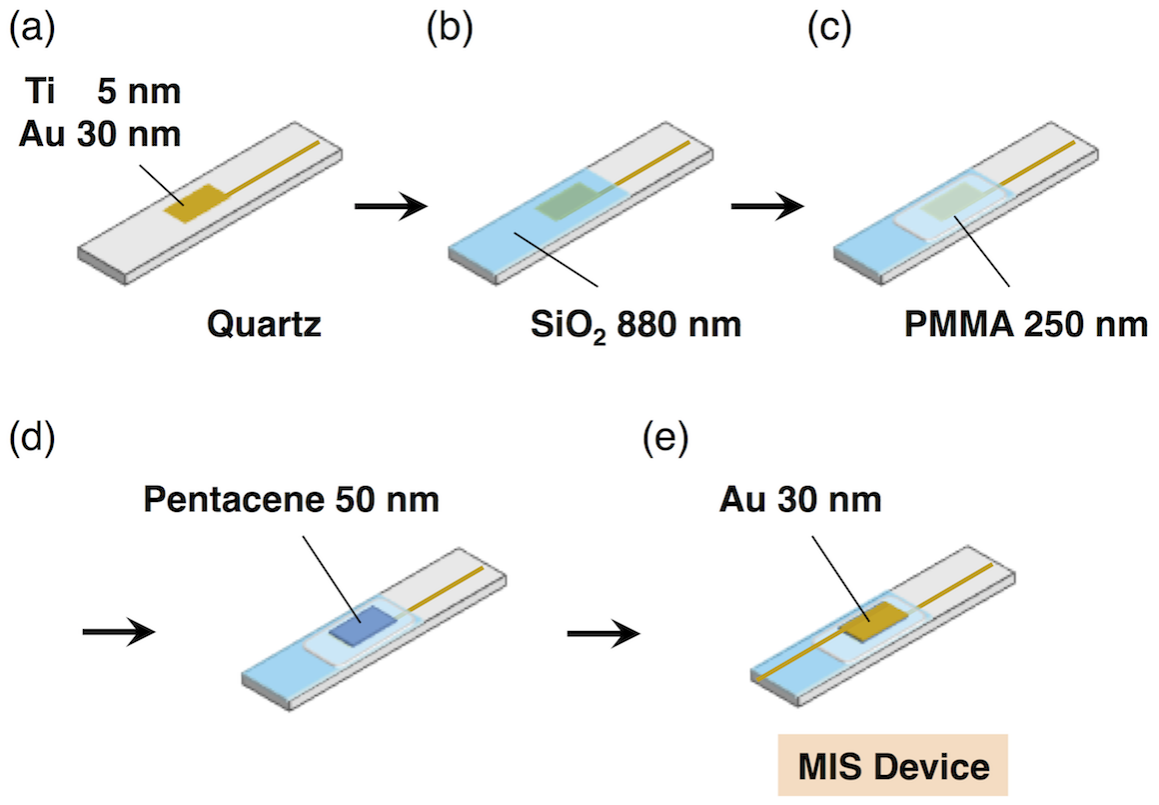
Schematic illustrations of the fabrication process of Metal-Insulator-Semiconductor (MIS) devices.

**Figure 3:**
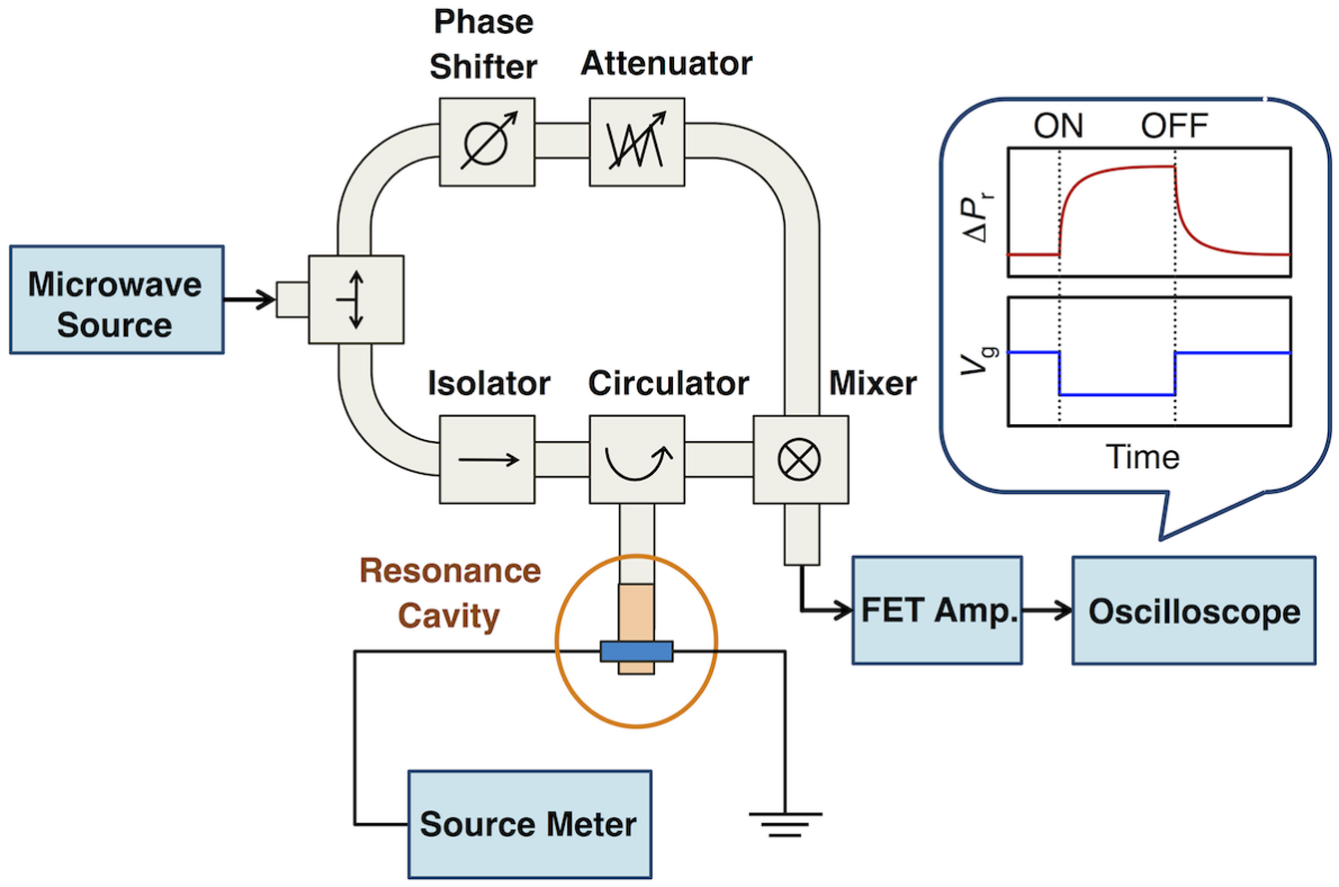
Schematic drawing of the FI-TRMC measurement system. The microwave circuit was designed for X-band microwaves (~9 GHz).

For the first demonstration of FI-TRMC, we prepared a metal-insulator-semiconductor (MIS) device adopting a Au/SiO_2_/PMMA/pentacene/Au configuration (Fig. 2). We used pentacene as a representative organic semiconductor with high hole mobility^25^,^26^, while PMMA was selected as an insulator. Two gold electrodes working as a gate enable control of the density and polarity of the accumulated charge carriers at the interfaces by applying a gate bias voltage, which leads to the direct quantification of the charge carrier numbers. Equation (1) shows the relationship between the change in charge carrier number (Δ*N*) and the change in the applied gate bias voltage (Δ*V*). 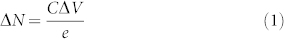
where *C* and *e* show the capacitance and the elementary charge of an electron, respectively. The value of *N* was determined by a Wayne Kerr Precision Component Analyzer 6430B capacitive sensor. Through atomic force microscopy (AFM), the vapor-deposited pentacene film in the MIS device (Fig. 2d) was characterized as having a typical polycrystalline morphology (Supplementary Fig. S1). In addition, an FET-type device with a configuration analogous to the MIS device (Fig. S2) exhibited typical FET characteristics (Fig. S3). These observations indicated that the MIS device is appropriately designed for FI-TRMC measurements. Fig. 3 shows a schematic illustration of a microwave circuit for FI-TRMC using X-band microwave radiation (~9 GHz). In FI-TRMC measurements, the microwave frequency and power are adjusted by a Rohde Schwarz SMF 100 A Signal Generator at ~ 9.0 GHz and 0.9–1.0 mW, respectively. The motion of charge carriers accumulated at the interfaces is modulated by the low electric field associated with the microwaves. Holes or electrons are directly injected by application of a microsecond-pulse bias voltage from a Wave Factory Multifunction Generator WF 1973, promoting field-induced charge-carrier generation at the pentacene–PMMA interface. The FI-TRMC signals picked up by a diode (rise time < 1 ns) were monitored by a Tektronix TDS5054 Digital Phosphor Oscilloscope. All the experiments were carried out under ambient atmosphere at room temperature.

### Principle of FI-TRMC method

When the MIS device is set in the microwave cavity (Fig. S4.) and a gate bias is applied, charge carriers are generated at the semiconductor/insulator interface. These carriers resonate as they absorb the incident microwaves which results in a change in the power of the reflected microwaves Δ*P*_r_. The relation between the change in the conductivity Δ*σ* (S cm^−1^) and the relative change in the reflected microwave power Δ*P*_r_/*P*_r_ is shown in equation (2)^27^. Equation (3) describes the relation between the change in the value of the electrical conductivity Δ*σ* caused by the applied gate bias and the change in the number of charge carriers Δ*N* that have accumulated in the MIS device due to the gate bias voltage. 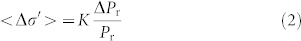


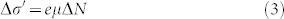
Here, *K* and *μ* denote an experimental parameter and the charge carrier mobility, respectively. In order to express the change in the reflected microwave power Δ*P*_r_ (W) in terms of Δ*Nμ* (cm^2^ V s), it is necessary to determine the quantitative relation between Δ*Nμ* and Δ*P*_r_.

### Correlation equation

**Figure 4:**
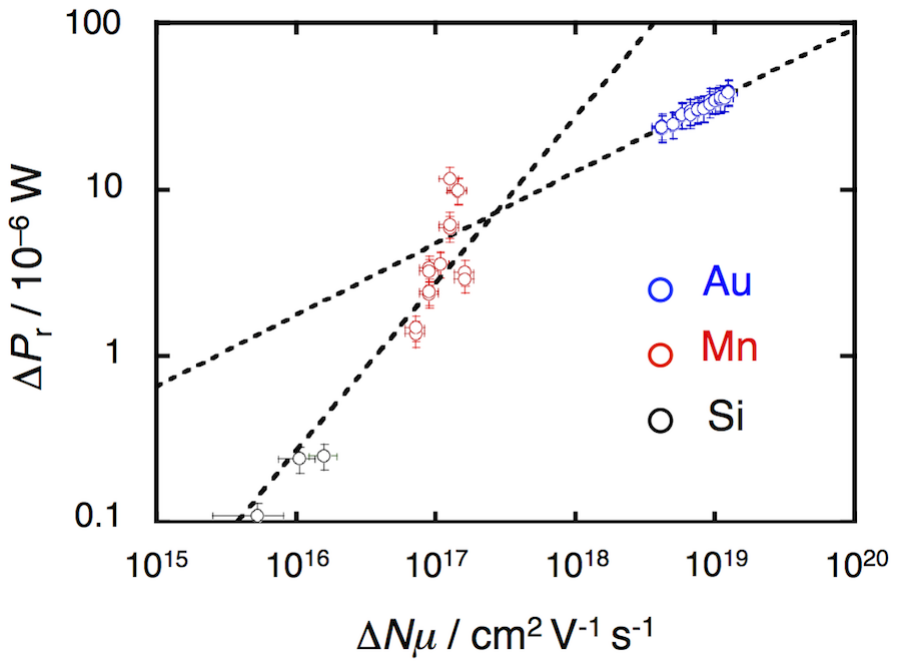
Correlation between the change of the reflected microwave power Δ*P*_r_ and the pseudo electrical conductivityΔ*Nμ* determined using the electrical conductivity of gold, manganese and silicon.

Prior to the measurement of the pentacene/PMMA MIS device, we investigated the relation between Δ*Nμ* and Δ*P*_r_ by loading standard samples into the microwave cavity. Gold (*σ* = 4.52 × 10^5^ S cm^−1^), manganese (*σ* = 6.94 × 10^3^ S cm^−1^), and *p*-doped silicon (*σ* = 10 S cm^−1^) were selected as the standard samples^28^. They were vapor deposited as thin films onto quartz substrates with their thicknesses controlled, so that the total deposited mass was known. The mass of these samples was then converted into the value of Δ*Nμ* using 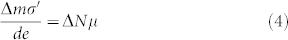
where *m* (g) and *d* (g/cm^3^) denote the mass and density of the standard samples, respectively. The value of Δ*Nμ* can be regarded as the change in the pseudo electrical conductivity. By measuring the value of Δ*P*_r_ for each standard sample, we obtained the relation between Δ*Nμ* and Δ*P*_r_ shown in Fig. 4. Two straight lines with different slopes were used to fit the data in the lower Δ*Nμ* (10^15^–10^17^) and higher Δ*Nμ* regions (over 10^18^). For lower Δ*Nμ*, the value of Δ*P*_r_ becomes small (*i.e.*: the change in the *Q* value is small), since Δ*P*_r_ is proportional to the electrical conductivity change (Δ*σ*)^29^,^30^. Thus, Δ*P*_r_ is proportional to the pseudo electrical conductivity Δ*Nμ*, and the data in the low Δ*Nμ* region (for Mn and *p*-doped Si) can be successfully fitted using the equation: 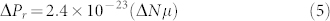
On the other hand, equation (5) does not provide a good fit to the data in the high Δ*Nμ* region (for Au). When samples with high electrical conductivity such as Au are used, the microwave amplitude decreases significantly at the surface of the thin film due to skin-depth effects. In such cases where the interaction of microwaves with a material is confined to the near-surface region, it is necessary to use the skin-depth approximation^31^: 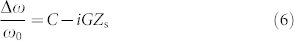


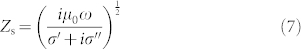
where Δ*ω* represents the difference in the resonance frequency between a cavity with and without a sample present, and *ω*_0_, *C*, *G*, *Z*_s_, and *μ*_0_ are the resonance angular frequency, the metallic shift associated with a perfect conductor, a resonator constant, the surface impedance, and the permeability, respectively. The *Q* value, representing the microwave power loss in the cavity, can be divided into *Q*_u_, *Q*_c_, and *Q*_s_ components associated with microwave interaction with the inner walls of the cavity, the coupling between the cavity and the waveguide, and the sample, respectively^29^. The *Q*_u_ and *Q*_c_ components are constant during the measurement of microwave power loss, so these can be included in *Q*_1_. The relation between *Q*_1_, *Q*_s_, and *Q* can be expressed as: 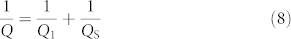
The relation between *Q*_s_, *ω*_0_, and the resonance frequency of the empty cavity *f*_0_ ( = *ω*_0_/2π) is expressed by^31^: 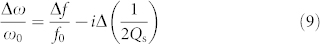
Microwaves passing through the sample give rise to a decrease in the electric field and a phase shift, as indicated by equation (9), and this causes a change in the *Q* value and resonant frequency. The intensity of the microwaves reflected from the cavity is expressed by equation (10): 
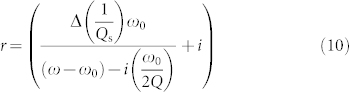
The value of Δ*P*_r_/*P*_r_ is defined by the complex conjugation of the reflectance: 
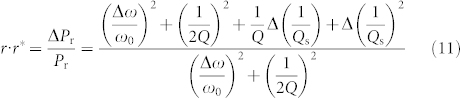
In the case of Δ*ω*/*ω*_0_ ~ 0, equation (11) can be approximated by a linear function of (1/*Q*_s_). 
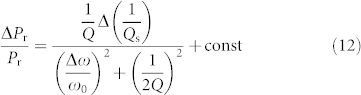
Then, substituting equations (6), (7) and (9) into equation (12) results in: 
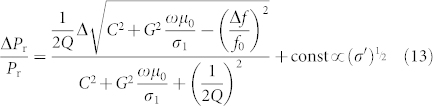
Remarkably, Δ*P*_r_ is proportional to (*σ*_1_)^1/2^. Therefore, the data for Au can be fitted using the following equation: 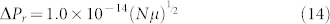
The values of *N* and *μ* for common organic semiconductors are smaller than those for Au, Mn, and Si. In fact, the value of *N* in MIS devices is known to be around 10^12^–10^13^ through calculation of their capacitance. Since the charge-carrier mobility for organic semiconductors is lower than 10 cm^2^ V^−1^ s^−1^, the value of Δ*Nμ* is 10^14^ at most, which allows us to conclude that the linear approximation shown in equation (5) sufficiently describes the relation between Δ*P*_r_ and Δ*Nμ*.

### FI-TRMC demonstration

**Figure 5:**
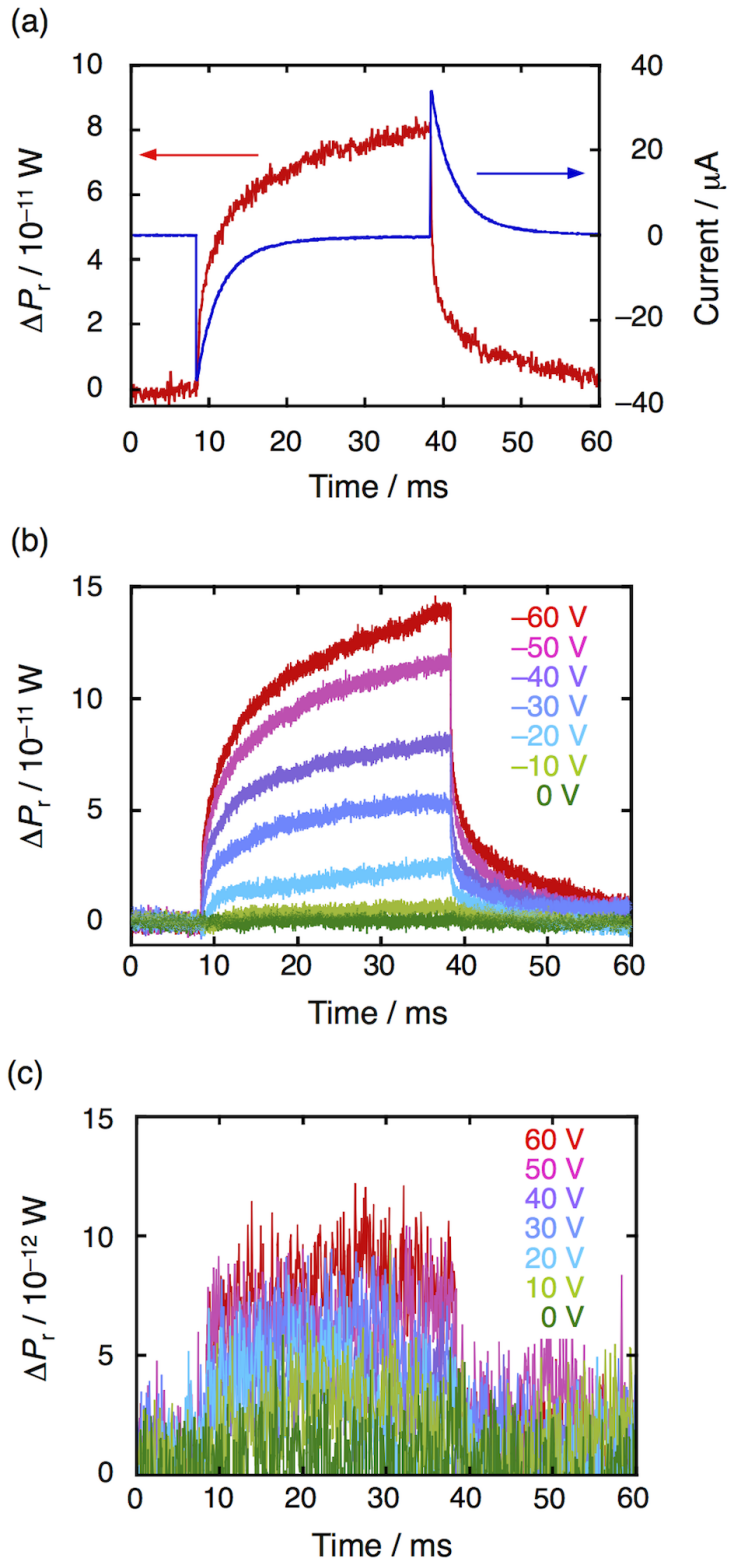
(a), Kinetic traces of FI-TRMC signal (red) and current flow (blue) detected in the pentacene/PMMA MIS device. A gate bias voltage of –40 V was applied from 9 to 39 ms. (b), Gate bias effect on FI-TRMC signals for holes. (c), Gate bias effect on FI-TRMC signals for electrons.

**Figure 6:**
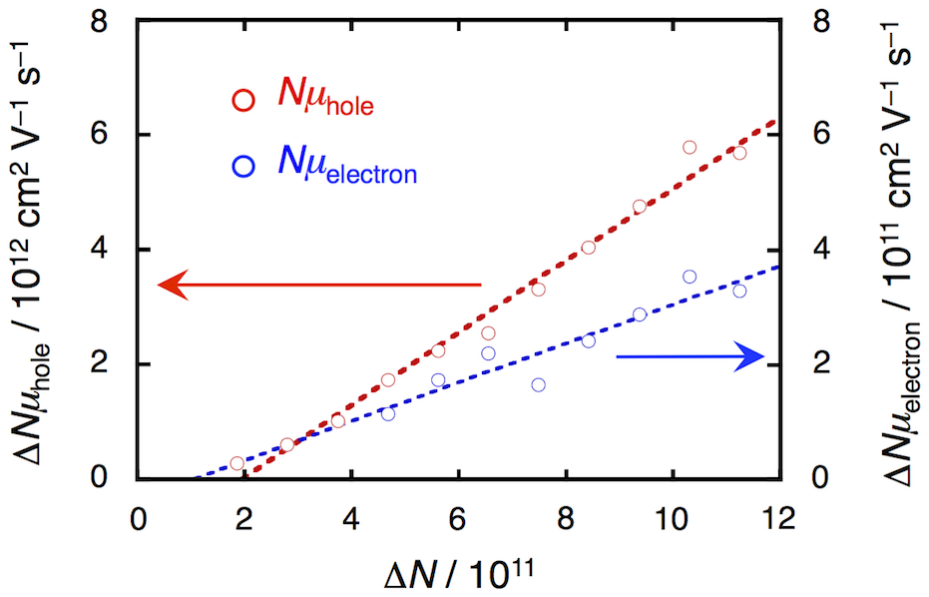
Correlation between charge carrier number Δ*N* and pseudo electrical conductivity Δ*Nμ*. Red and blue circles indicate holes and electrons, respectively. The slopes of the fitted curves correspond to the value of the mobility *μ*.

When a gate bias of –40 V was applied, the pentacene/PMMA MIS device displayed a time-dependent microwave power change (Δ*P*_r_) with saturated behavior (Fig. 5a), where the bias voltage was applied between 9 to 39 ms. At the same time, the charging-discharging current appeared to respond to the applied gate bias (Fig. 5a). The differential of Δ*P*_r_ was in good agreement with the current signal, which indicates that the Δ*P*_r_ response is associated clearly with charges accumulated in the MIS device. The Δ*P*_r_ response resembles the voltage response characteristics of a capacitor in a typical resistor–capacitor (RC) circuit, and in the decay region (39–50 ms) gives the linear plots shown in Fig. S5. Fig. 5b. shows the dependence of Δ*P*_r_ on the applied gate bias. When the applied gate bias voltage was decreased from 0 V to –60 V, Δ*P*_r_ increased proportionally, probably because the number of accumulated holes became large in relation to the absolute value of the gate bias. In sharp contrast, the response of Δ*P*_r_ to the positive gate bias was poor from 0 V to 60 V (Fig. 5c), suggesting a lower conductivity for electrons in the MIS device. After measuring Δ*P*_r_ at each gate bias and capacitance, and using equations (5) and (1), we can draw the Δ*N*–Δ*Nμ* plots, as shown in Fig. 6. The slope ( = δ*Nμ*/δ*N*) corresponds to the value of the charge carrier mobility, providing the approximate values *μ*_hole_ = 6.3 cm^2^ V^−1^ s^−1^ and *μ*_electron_ = 0.34 cm^2^ V^−1^ s^−1^ for the pentacene MIS device.

## Discussion

There are many reports on FET hole mobilities of over 1 cm^2^ V^−1^ s^−1^ in single pentacene crystals^32^,^33^, where the value is considered to be the boundary between hopping and band conduction mechanisms for charge carrier transport^34^,^35^,^36^. Considering the nanometer-scale spatial motion of charge carriers in the FI-TRMC measurement, the range of motion of holes is assumed to be within a single domain. Therefore, the high hole mobility of 6.3 cm^2^ V^−1^ s^−1^ suggests that the transport mechanism for holes involves band conduction within a domain. It should also be noted that precise evaluation of hole mobility in pentacene by the FP-TRMC method has been limited so far, due to the lack of photo-induced charge carriers^37^. In that sense, the present work provides the first evaluation of intrinsic hole mobility in pentacene materials. Meanwhile, there have been no reports on the electron transport properties of pentacene thin films detected directly in air. The reason for electron transport occurring in air, as observed in the present work, is most likely due to the local motion of electrons. This in turn means electrons can avoid traps such as oxygen molecules, impurities, and crystal grain boundaries in a pentacene thin solid film, also suggesting that such traps dramatically inhibit the long translational motion of electrons.

Overall, we successfully developed the FI-TRMC measurement system for evaluating charge carrier mobility including the effect of the interfaces and minimizing the effect of grain boundaries. Considering nanometer-scale charge carrier motion and a hole mobility as high as 6.3 cm^2^ V^−1^ s^−1^, we clarified that hole transport occurs via a band conduction mechanism within individual pentacene domains. The value of hole mobility measured by FI-TRMC is as high as the FET hole mobility in a single pentacene crystal^32^,^33^, which potentially allows us to determine the charge carrier mobility in single crystalline states even using polycrystalline materials. Considering the above achievements, we believe that FI-TRMC, the newly-developed measurement system demonstrated here, serves as not only an potential evaluation system for FET mobilities in thin-film materials, but also a powerful tool for investigating the effect of insulating materials on the charge carrier mobility.

## Methods

### Device fabrication

Pentacene and poly(methyl methacrylate) (PMMA) were purchased from Aldrich. The quartz substrate (4.9 mm × 50 mm) was treated by UV–O_3_ prior to use. For fabrication of the gate electrode, Ti (3 nm) and Au (30 nm) were deposited on the substrate through a shadow mask in a vacuum deposition system. The interaction between the probing microwave and ultra-thin metal electrodes is expressed by the skin depth (*δ*) equation: 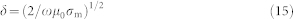
where *ω*, *μ*_0_, and *σ*_m_ are the frequency of interacting microwave, magnetic permeability, and conductivity in the metal electrode, respectively. The use of 10 GHz microwave and Au electrodes results in its value of *δ* as ~ 790 nm, suggesting the thin enough thickness of the metal electrodes (5 and 30 nm) to unveil the organic semiconductor layer from the metal electrode shielding. The area of the gate electrode was 3 mm × 6 mm. The substrate was then set in a vacuum deposition system equipped with a RF-sputtering gun under dry Ar gas at room temperature. The SiO_2_ gate insulator was deposited by RF-sputtering at 100 W onto the Au gate electrode through a shadow mask under an Ar gas flow at 1.1 Pa. The thickness was approximately 880 nm. A PMMA dielectric layer was spin-coated onto the substrate at 250 nm with 3 wt% PMMA in a toluene solution and annealed at 100°C under N_2_ gas. A pentacene organic semiconductor layer was deposited onto the substrate to 50 nm in thickness by vacuum deposition under 4–6 × 10^−5^ Pa. The rate of deposition was 0.3–0.5 Å per second. After the deposition of the pentacene, a 30 nm thick Au layer was deposited through a shadow mask to fabricate the source electrode.

## Author Contributions

S.S. conceived and designed the research. Y.H. and T.M. performed experiments. Y.H., T.M., T.S., A.S. and S.S. analyzed the data and wrote the manuscript. All authors read and discussed it extensively.

## Supplementary Material

Supplementary Information Supplementary Infomation

## Figures and Tables

**Figure 1:**
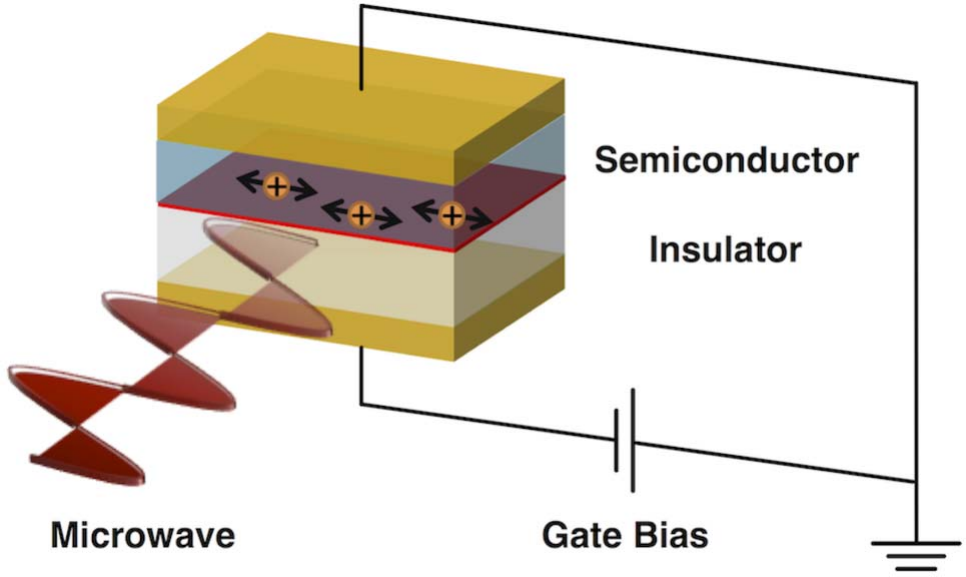
Conceptual illustration of field-induced time-resolved microwave conductivity (FI-TRMC) technique.
