# Supplementary material for: Evaluation of Intrinsic Charge Carrier Transport at Insulator-Semiconductor Interfaces Probed by a Non-Contact Microwave-Based Technique
Source: Sci Rep. 2013 Nov 11;3:3182. doi: 10.1038/srep03182 (PMC3822380; doi:10.1038/srep03182)
Supplement: Supplementary Information — Supplementary Infomation [file srep03182-s1.doc]

**Supplementary Information**

**Evaluation of Intrinsic Charge-Carrier Transport in Insulator-Semiconductor Interfaces Probed by Non-Contact Microwave-Based Technique**

Yoshihito Honsho, Tomoyo Miyakai, Tsuneaki Sakurai, Akinori Saeki, and Shu Seki*

*Department of Applied Chemistry, Graduate School of Engineering, Osaka University, 2-1 Yamadaoka, Suita, Osaka 565-0871, Japan*

E-mail: seki@chem.eng.osaka-u.ac.jp


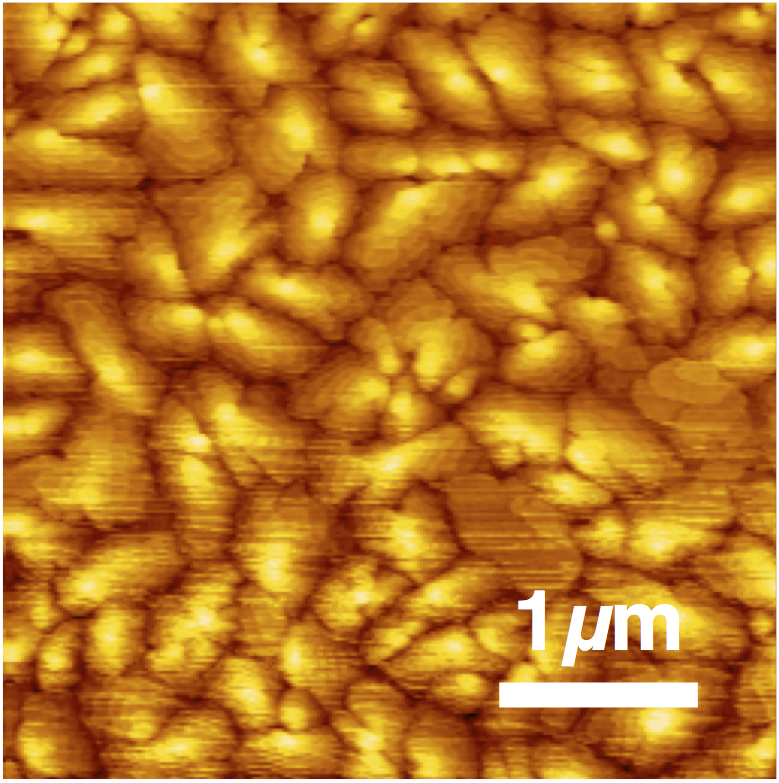


***Figure S1.*** AFM image of the pentacene film in the MIS device.


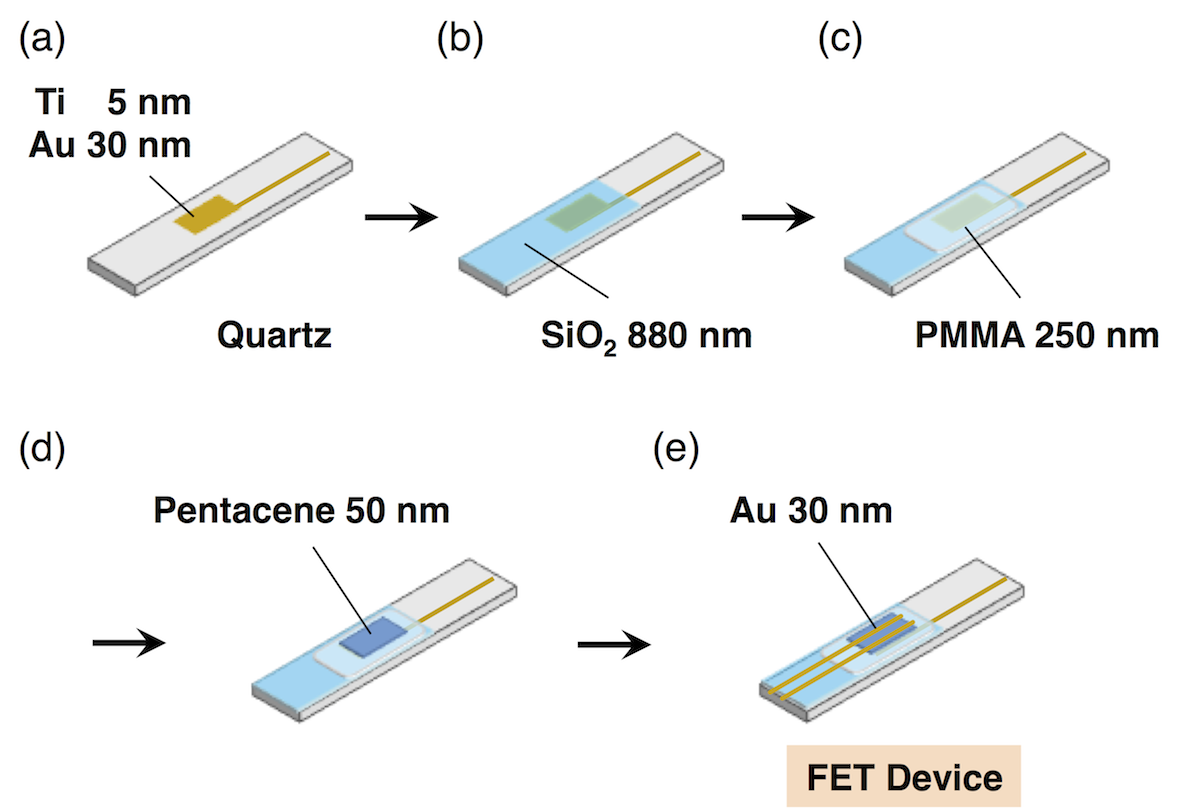


***Figure S2.*** Schematic illustrations of the fabrication process of FET devices.


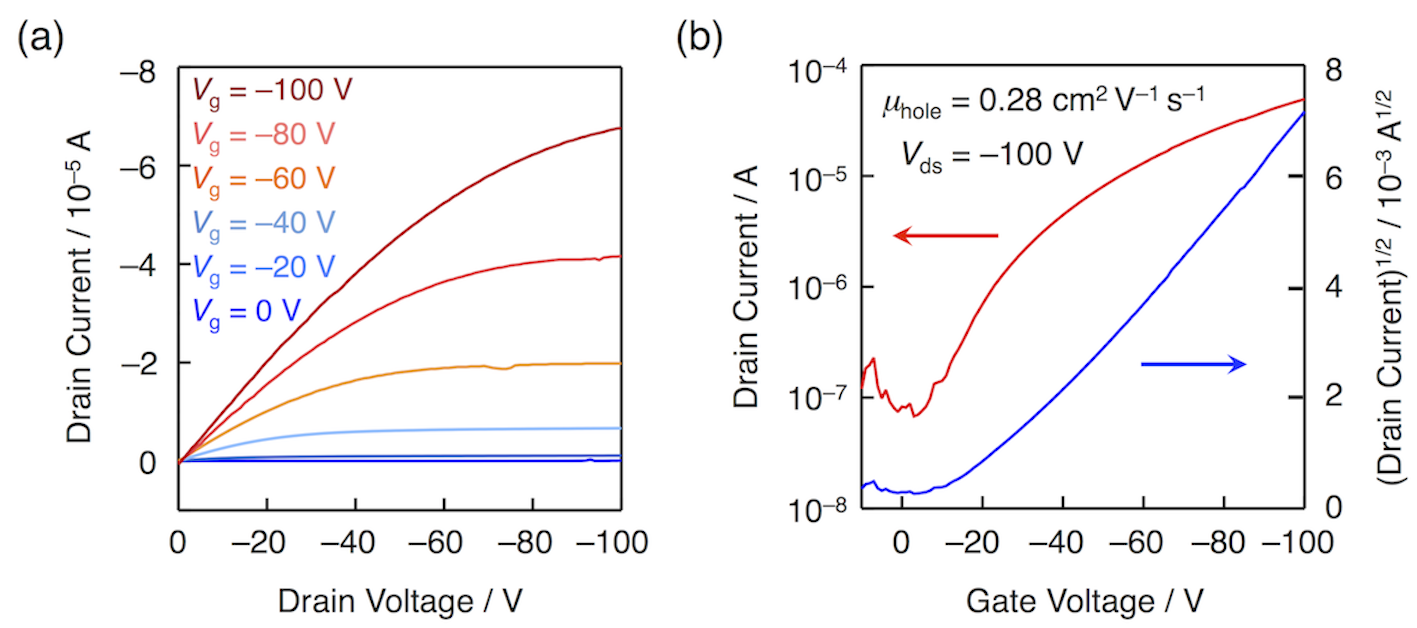


***Figure S3.*** (a) Output and (b) transfer characteristics of the pentacene-based FET device.


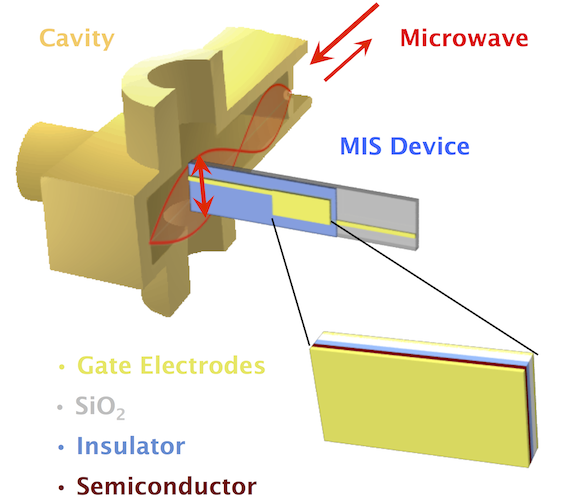


***Figure S4.*** Conceptual illustration of a MIS device in the resonance cavity.


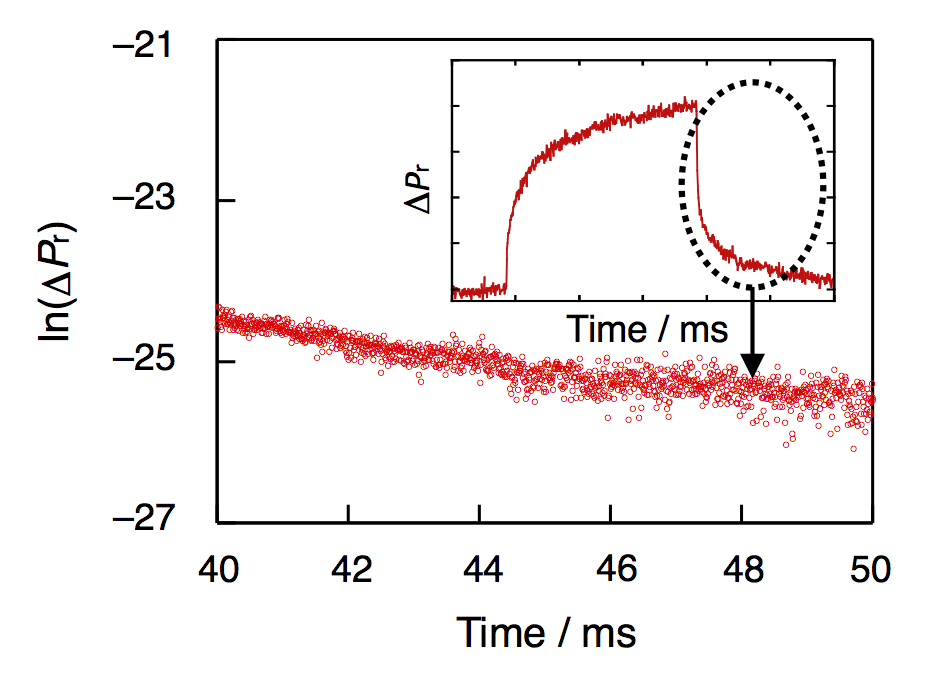


***Figure S5.*** Logarithm plots of FI-TRMC decay signal. Inset shows the linear plots identical to the red curve in Figure 5a.


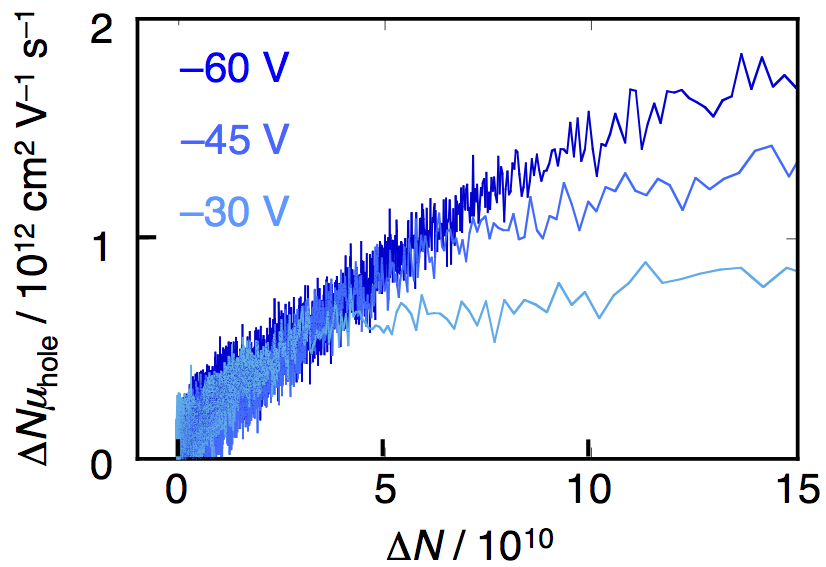


***Figure S6.*** Correlation plots between charge carrier number Δ*N* and pseudo electrical conductivity Δ*Nμ* for the discharging process of the pentacene device with applied gate biases at –30, –45, and –60 V. Δ*N* and Δ*Nμ* values in each time were determined from the current flows and combination of reflected microwave power and equation (5), respectively. The observed linearity indicates the time-dependent discharging behavior with a constant *μ *value. The nonlinear region observed for the earlier time range originates from the different instrumental functions.


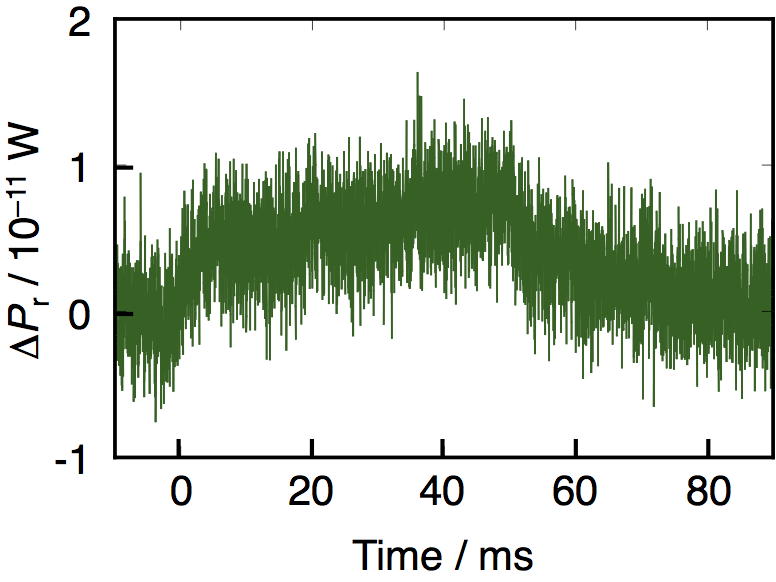


***Figure S7.*** Kinetic traces of FI-TRMC signal for electrons detected in the C_60_/PMMA MIS device. Thickness of C_60_ layer was 75 nm. A gate bias voltage of +150 V was applied from 0 to 50 ms.
